# Using ultrasonography for verifying feeding tube placements in cats

**DOI:** 10.3389/fvets.2023.1220547

**Published:** 2023-11-29

**Authors:** Barbara Bruno, Paolo Savarino, Renato Zanatta, Silvia Rallo, Andrea De Giovanni, Cristiana Maurella, Antonio Borrelli

**Affiliations:** ^1^Department of Veterinary Science, University of Turin, Torino, Italy; ^2^Istituto Zooprofilattico Sperimentale del Piemonte, Liguria e Valle d’Aosta, Torino, Italy

**Keywords:** feeding tubes, cats, PoCUS, intensive care units, ultrasound

## Abstract

**Introduction:**

This study aimed to investigate the use of ultrasonography for verifying feeding tube placement in hospitalized cats compared with radiographic evaluation.

**Methods:**

This prospective investigation was performed on client-owned cats. The position of the feeding tube was checked using right lateral thoracic radiography and ultrasonography. Ultrasound examinations were performed using a high-frequency linear transducer and a microconvex transducer. The examination was performed in three steps: transverse and longitudinal planes of the left side of the animal’s neck to identify the feeding tube in the esophagus, and a longitudinal angled plane of the epigastrium to identify the tube at the lower esophageal sphincter.

**Results:**

A total of 25 cats were included in this study. Assessing the correct positioning of the feeding tubes using a right lateral thoracic radiograph revealed that the tube was in the distal esophagus in 12/25 cats and reached the stomach in 13/25 cases.

**Discussion:**

In all cats, both ultrasonography and right lateral chest radiography identified the feeding tube at the esophageal level. For stomach, ultrasonography demonstrated good values of diagnostic performance compared to radiography, with excellent reliability and validity in terms of sensitivity and predictive value. Ultrasonography is a valid tool for confirming tube placement in the esophagus and is almost as efficient as radiology.

## 1 Introduction

Feeding tube insertion (nasogastric or nasoesophageal feeding tube) is a common procedure performed in veterinary intensive care units for gastric decompression and content removal or administering enteral nutrition and medications in anorexic animals.

These tubes can be easily placed and readily used, often with minimal or no sedation. Although this technique is relatively simple, several complications associated with mispositioning have been reported in the literature, such as insertion in the tracheobronchial tree, pneumothorax, pneumomediastinum, subcutaneous emphysema, pneumonia, pulmonary haemorrhage, empyema, haemothorax, bronchopleural fistula, mediastinitis or perforation of the esophagus, and, in rare cases, even intravascular or intracranial malpositioning (1–9).

Methods to determine correct feeding tube placement include measuring tube length, observing aspiration of gastric contents, infusing 3–5 mL of sterile saline through the tube, observing cough response, aspirating gastric contents, determining pH or bilirubin, applying capnography, or obtaining a chest radiograph (10, 11). Although sampling of carbon dioxide with capnography from the feeding tube during tube insertion can verify incorrect tube placement in the trachea, it cannot differentiate between esophageal or gastric placement (12). Presently, confirmation using radiographic evaluation is the gold standard method of diagnosis in human and veterinary medicine; however, in human medicine, concerns about radiation exposure have led to the investigation of alternative methods to detect correct tube placement (1, 2, 5, 7, 13).

Recently, the use of point-of-care ultrasound has become widespread in human emergencies and in veterinary medicine. It is performed by non-ultrasound specialists and is a rapid, noninvasive, and time-saving assessment of anatomical structures and pathological conditions, contributing to improved patient care (13–15). Owing to its numerous advantages (wide availability, easy applicability, possibility of repeat evaluations, bedside assessment, rapidity, cost-effectiveness, absence of ionizing radiation, and possibility of obtaining dynamic images), point-of-care ultrasound is currently used by emergency physicians to verify feeding tube placement in adult and pediatric patients, particularly in resuscitation room or in intensive care units when other methods of confirmation are not readily available (4, 16–18).

In this prospective study, we aimed to investigate the use of ultrasound in hospitalized cats to verify feeding tube insertion into the gastrointestinal tract, by comparing ultrasound and radiographic evaluations.

## 2 Materials and methods

This was a prospective investigation of client-owned cats. The study protocol was approved by the Institutional Ethics and Animal Welfare Committee (protocol no. 1128). The owners were informed of the study protocol and each one of them signed an informed consent form accordingly.

Cats admitted to the intensive care unit of the University Veterinary Hospital were included in the study if they required the placement of a nasogastric tube for nutritional support or aspiration of gastric contents. The following characteristics were considered as exclusion criteria: severe facial trauma, nasal discharge, hemostatic disorders, thrombocytopenia, soft tissue trauma, or severe skin lesions of the neck or abdomen that would affect the ultrasound evaluation.

### 2.1 Placement of feeding tube

The feeding tube was made of polyvinyl chloride (Vygon, 6 Fr, 400 mm). Tube insertion was performed by an emergency physician by measuring the distance from the nostril to the level of the ninth rib (landmark for placement into the distal esophagus) or 13th rib (landmark for placement into the stomach) and marking the tube at the required length using butterfly tape or a permanent marker pen. The patient was placed in sternal recumbency on an examination table. A local anesthetic was applied to the nostril (0.1 mL lidocaine 2%) and the tip of the tube was lubricated with 1% lidocaine hydrochloride gel. The head was restrained and slightly elevated, and the tube was inserted into the nostril in the ventral and medial directions, while simultaneously observing the signs of swallowing, which indicated that the tube was correctly directed towards the esophagus, or coughing and discomfort, which indicated that the tube may have been inserted into the trachea. When the marked length was reached, the physician attached a 5 mL syringe to the end of the feeding tube and aspirated it back to check for negative pressure or aspiration of gastric content. The tube was then secured to the nose and cheek using adhesive tape with glue or sutures, and an Elizabethan collar was placed to prevent displacement of the tube by the cat.

After placement, the tube position (nasogastric or nasoesophageal feeding tube) was checked by obtaining a right lateral thoracic radiograph, followed by ultrasonography. All radiographs included neck and retro diaphragmatic region and tubes had to be identify in the region of the pharynx, dorsal to the trachea and above the tracheal carina; when nasogastric feeding tube was placed, the tip of tube need also to be properly detected in stomach. Ultrasonography was performed in the ICU by an operator who was not involved in the radiographic evaluation. The ultrasound examinations were blinded to the location of the feeding tube (nasogastric or nasoesophageal) and the results of prior tests performed after tube insertion.

### 2.2 Ultrasonography

**Figure 1:**
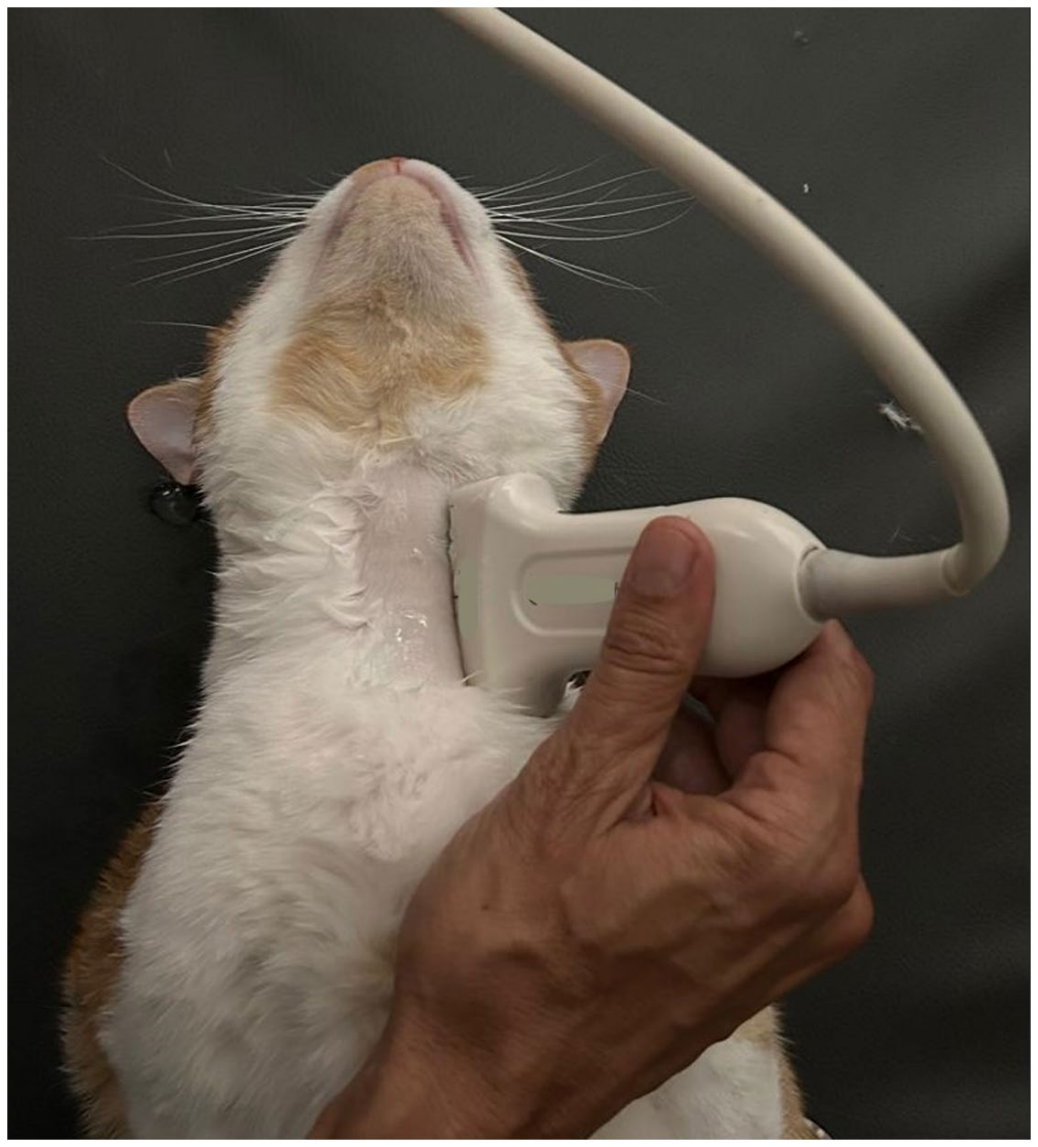
Longitudinal plane of the left side of the cat’s neck with a high-frequency linear transducer, to identify the feeding tube into the esophagus.

**Figure 2:**
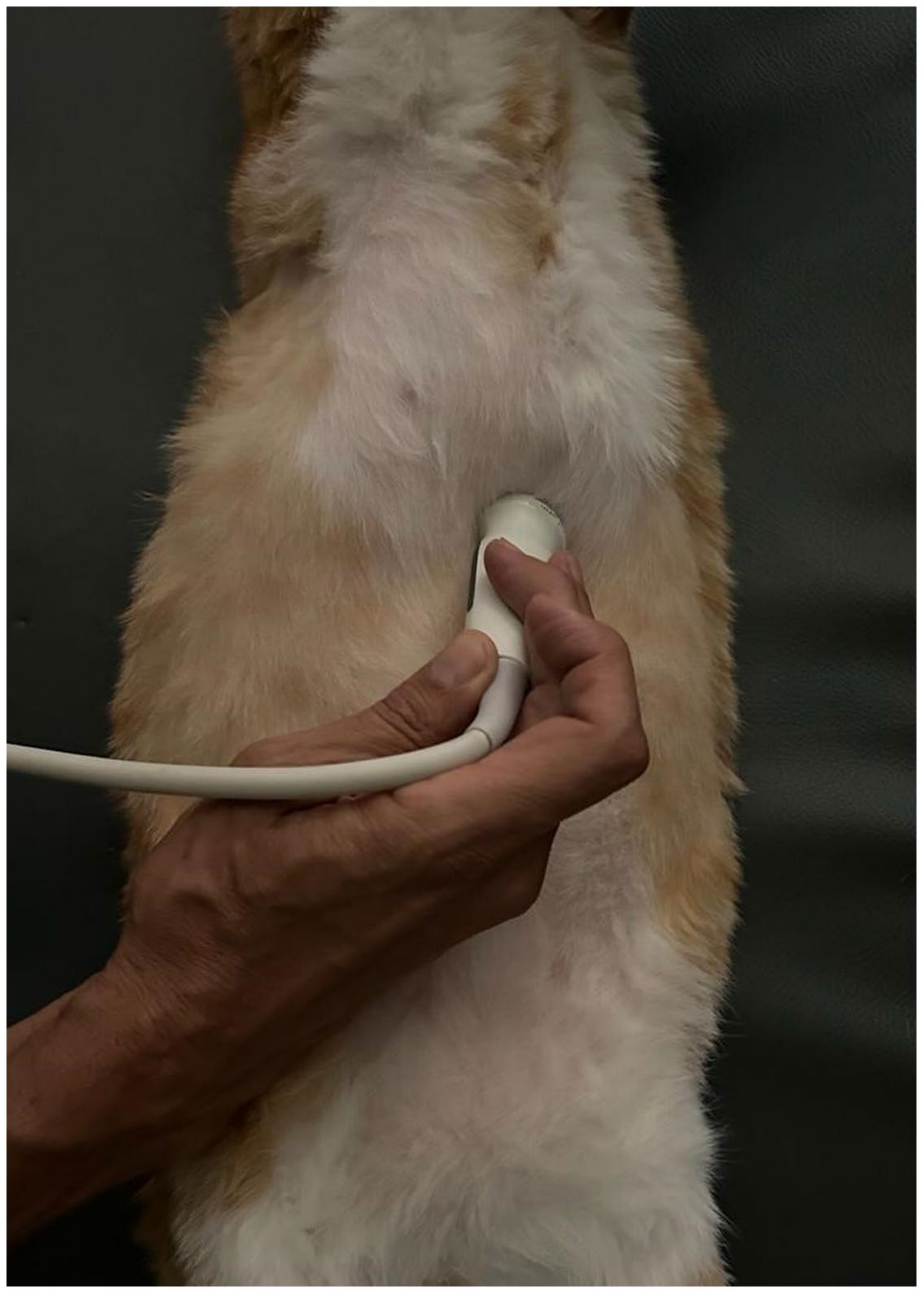
Longitudinal angled scan of the epigastrium with a microconvex transducer, to identify the tube at the esophageal sphincter. The probe was slightly parasagittal and oriented towards the left cranial abdominal quadrant.

**Figure 3:**
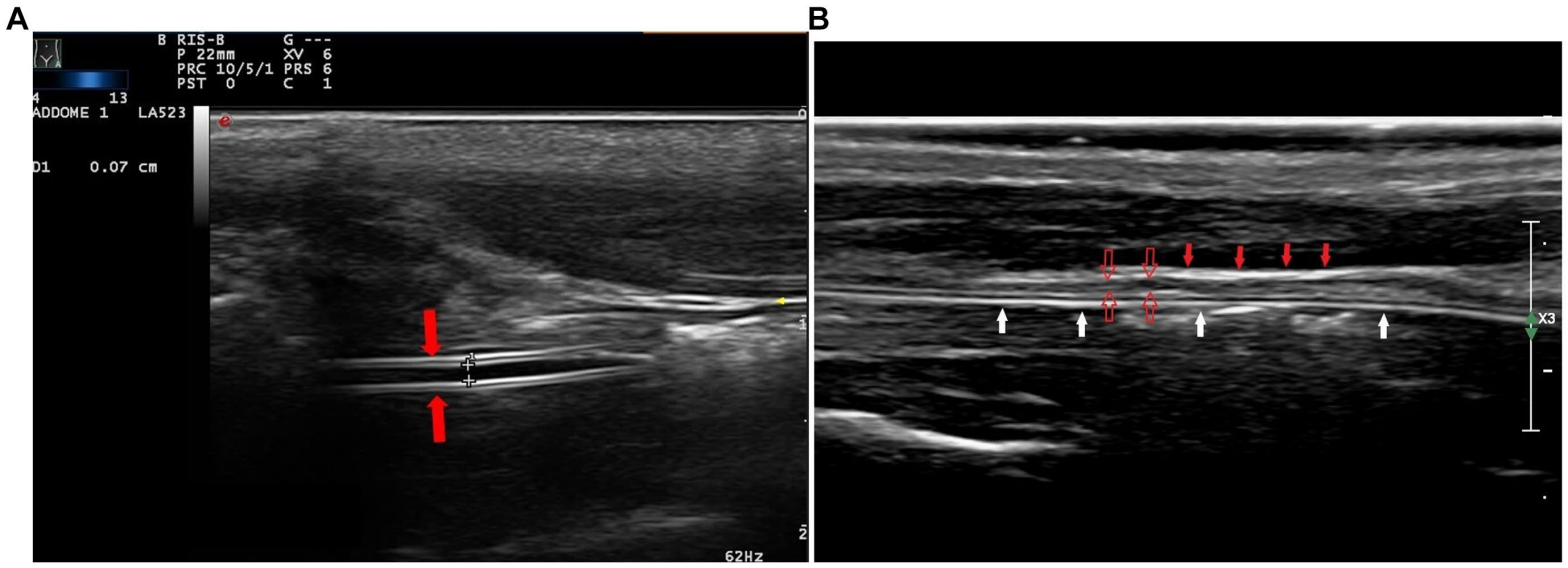
In the longitudinal cervical scan, with a high-frequency linear transducer, the feeding tube was visible as double parallel hyperechogenic lines (**A**, red arrows). The hyperechoic adventitia (**B**, full red arrows) and the hypoechoic muscularis (**B**, empty red arrows) are the most evident layers of the walls of the esophagus; the tube is within the lumen (**B**, white arrows).

**Figure 4:**
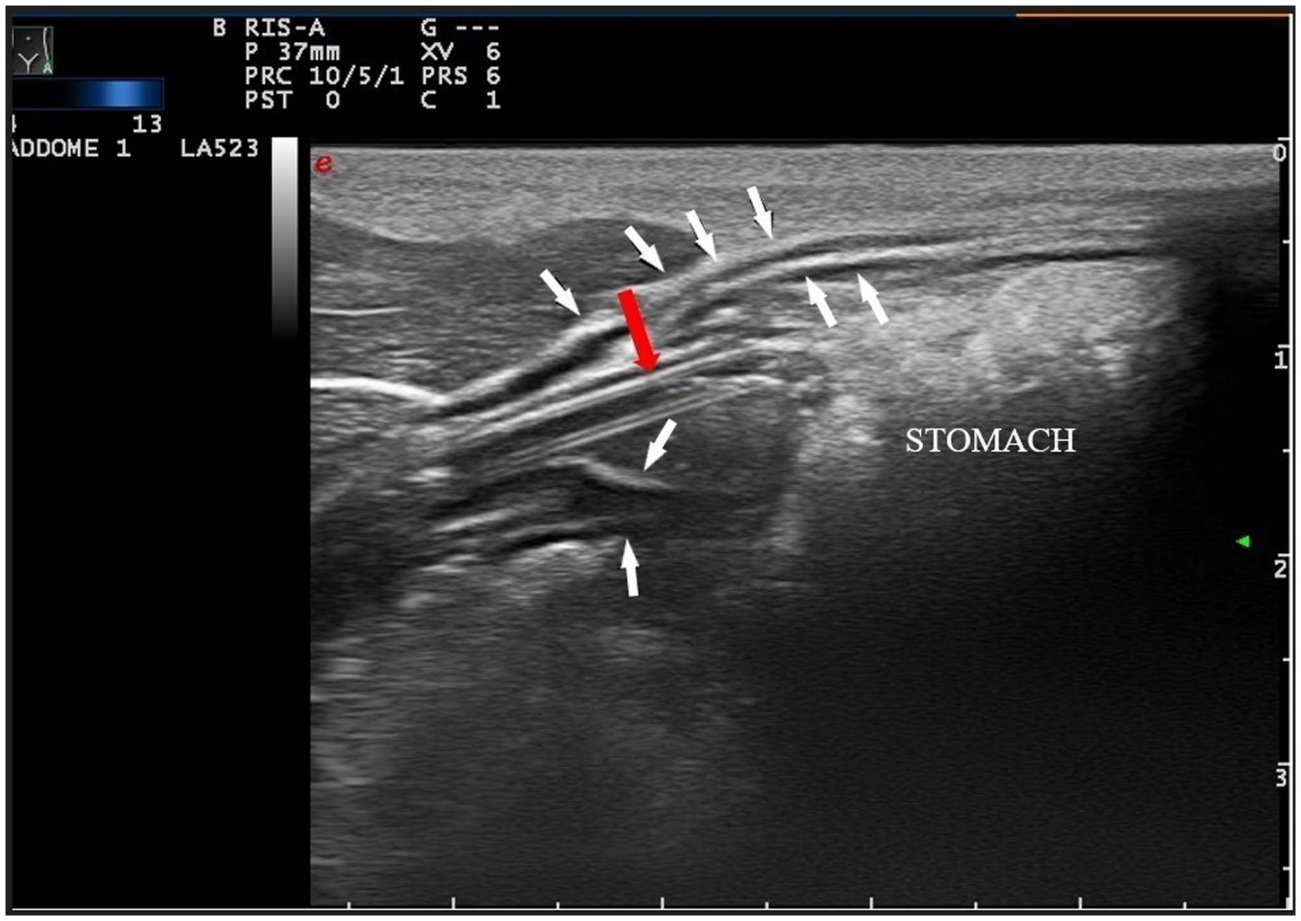
Longitudinal angled plane of the epigastrium with a high-frequency linear transducer. The feeding tube (pointed by the red arrow) was identified as a double parallel hyper-echogenic line within the stomach lumen (white arrows indicate the gastric wall). To improve the quality of the picture, a linear probe has been used, instead of a microconvex probe.

Ultrasound examination (Esaote MyLab 70 Xvision) was performed by veterinarians specialized in ultrasound (RZ, PS, and AD), using a high-frequency linear transducer (4–13 mHz) and a microconvex transducer (3–9 mHZ). The examination was performed in three steps: transverse and longitudinal plane of the left ventrolateral side of the animal’s neck to identify the feeding tube in the distal esophageal sphincter (Figure 1) and a longitudinal angled scan of the epigastrium to identify the tube at the esophageal sphincter (Figure 2). First, the patient was placed in sternal recumbency with a raised head, and a high-frequency linear probe was placed transversely on the ventral surface of the neck, just below the cricothyroid membrane, towards the left side. The cervical esophagus appeared as an oval structure in the left paratracheal space on the transverse scan and as a long tubular structure on the longitudinal scan. Each wall of the esophagus has five layers, hyperechoic adventitia, hypoechoic muscularis, hyperechoic submucosa, hypoechoic mucosa and hyperechoic mucosal surface (19). If the tube was present in the esophagus, a hyperechoic structure with reverberation was visible in the transverse scan, and a double parallel hyperechogenic line was visible in the longitudinal scan (Figure 3). Subsequently, with the patient in dorsal recumbency, the microconvex probe was placed in the subxiphoid area, slightly parasagittal, and oriented towards the left cranial abdominal quadrant to identify the distal esophageal sphincter. Normally, the gastric cardia is visible between the liver and abdominal aorta. A positive finding was defined as identification double parallel hyperechogenic lines within the stomach (Figure 4).

To verify that the structure identified in the esophagus was the tube and confirm the location of the tube in the esophagus, an additional assessment was performed by injecting a mixture of 4 mL of saline and 1 mL of air, agitated to make microbubbles (bubble contrast), through the feeding tube and concurrently examining for flow in the longitudinal plane of the cervical part of the esophagus and for detection of hyperechogenic “fog” in the stomach.

### 2.3 Statistical analysis

The number of subjects required was calculated as to test the *H*_0_: *ρ*_1_ = *ρ*_2_, *α* = 0.05 (two-sided) and power = 80% (20). To assess the reliability between the two methods used, Cohen’s kappa was calculated; when all the samples showed the same result (i.e., all positives) and the Cohen’s kappa was not calculable the agreement was evaluated. Furthermore, using the radiography as Gold Standard, the accuracy of the ultrasonography compared to radiographic evaluation was calculated in terms of sensitivity and specificity. All analyses were performed using Stata 17.1.

## 3 Results

A total of 25 cats were included in this study. The study population consisted of 12 spayed females and 13 neutered males with a median age of 8 years (min. 2-max. 18), the median body weight was 4.6 kg (min. 1.7-max. 10), and the breeds included European shorthairs only (*n* = 25). The underlying diseases of the cats were: chronic kidney disease (*n* = 3), gastroenteritis (*n* = 3), intestinal neoplasm (*n* = 3), urethral obstruction (*n* = 2), polytrauma (*n* = 2), cholangiohepatitis (*n* = 5), pancreatitis (*n* = 2), intestinal foreign body, diaphragmatic hernia, immune-mediated hemolytic anemia, thymoma, and pulmonary neoplasm (1 cat from each cause).

The feeding tubes were inserted without complications, and none were displaced into the trachea. Accordingly, accuracy and reliability were calculated on the ability to identify the tube in the esophagus and stomach.

Assessing the correct positioning of the feeding tubes using right lateral thoracic radiography allowed the identification of tube in esophagus in 25/25 cats; in particular 12/25 were nasoesophageal feeding tubes and 13/25 were nasogastric tubes. In all cats, both ultrasonography and right lateral chest radiography have identified the feeding tube at the esophageal level (25/25) showing a maximum level of agreement between the two methods (100%, CI 95% 86–100). Tubes were detected within the gastric lumen by ultrasound in 16 cases, but nasogastric tubes identify by radiography were actually 13/25. In 3 cats, ultrasound then incorrectly showed the tube within stomach (false positive identification), revealing a lower value of Cohen’s kappa (*k* = 0.76, CI 95% 0.49–1) (Table 1).

**Table 1:** Repeatability of the ultrasound and of the bubble contrast compared to radiography.

ESOPHAGUS			Agreement	CI 95%
Longitudinal plane	vs	Radiography of the esophagus	100%	(86–100)
Transverse plane			96%	(88–100)
Bubble contrast			92%	(81–100)

STOMACH			Cohen’s kappa	CI 95%
Stomach plane	vs	Radiography of the stomach	0.76	(0.49–1)
Bubble contrast			0.26	(−0.1–0.64)

CI, confidence interval.

Considering radiography as the gold standard in identifying the presence of the feeding tube, we evaluated the accuracy of ultrasound both at esophageal level and gastric levels. The feeding tubes in the esophagus were visible on ultrasonography in all patients, resulting in a sensitivity of 100%. When the tube did not reach the stomach in the right lateral thoracic radiograph, ultrasonography correctly assessed the position in the esophagus identifying the tube at the level of the neck and not detecting the tube in the gastric lumen. Furthermore, ultrasonography showed a good capability to identify when the tube reached the stomach (Se = 100%) but identified also 3 feeding tubes as within the stomach which actually were not (false positives, Sp 75%) (Tables 2, 3).

**Table 2:** Accuracy of ultrasonography compared to the radiography at esophagus level.

Number of correct positions identified by X-ray	Number of correct positions identified by US	Sensitivity
25	25	100% (CI 95% 86.3–100)

Specificity was not calculable as all the feeding tubes were correctly positioned and identified. X-ray, radiography; US, ultrasound; CI, confidence interval.

**Table 3:** Accuracy of ultrasonography compared to the radiography at stomach level.

		Position confirmed by X-ray			
		Nasogastric (*n* = 13)	Nasoesophageal (*n* = 12)		
Positions identified by US	Nasogastric (*n* = 16)	13	3	Sensitivity 100% (CI 95% 75–100)	Specificity 75% (CI 95% 43–95)
	Nasoesophageal (*n* = 9)	0	9		

X-ray, radiography; US, ultrasound; CI, confidence interval.

The results derived from the application of bubble contrast into the feeding tubes showed that in 2/25 cats, the contrast was not identified in the longitudinal planes of the esophagus, and in 6/25 cases, it was not identified in the stomach projection, reducing the level of agreement of ultrasound (Table 1).

## 4 Discussion

To avoid complications, the position of the feeding tube in the esophagus or stomach must be confirmed quickly and accurately before initiating administration of food to avoid complications. In human medicine, current guidelines recommend radiography as the gold standard method for establishing feeding tube location; however, other ancillary techniques, such as evaluation of pH in gastric aspiration fluids, capnography, and ultrasound technology, have also been reported in literature (11, 21). Ultrasonography is promising and potentially useful since it is a noninvasive technique that avoids radiation exposure.

Analysis of the results obtained in the present study showed that ultrasonography has good agreement compared to radiography, suggesting that the technique has obtained good diagnostic performance in predicting feeding tube placement in the esophagus, resulting as effective as radiographic evaluation. Detection of feeding tubes in the stomach is less sensitive. When the tube was not identified with ultrasound in the gastric lumen, there was a high chance that it did not reach the stomach, when the same position was checked with radiography, but in 3 cases the feeding tubes were incorrectly identified by ultrasound within the gastric lumen. Identification of the feeding tube in the stomach using ultrasound may be affected by different conditions of gastric filling. For example, the presence of gas or food limits the identification of structures inside the gastric lumen, whereas the liquid is hypoechoic, making it easier to identify the hyperechoic tube. An explanation for the generation of false-positive results could be the identification of tubes in the esophagus, near the cardia, and the presence of gastric folds in the empty stomach, which simulate a small linear structure. In the case of an empty stomach, once the tube has been identified to be in the esophagus, it may be helpful to introduce a small amount of water into the stomach to create improve the visibility of structures inside the lumen.

No feeding tubes were misplaced in the trachea, which made it difficult to interpret how ultrasonography was useful for detecting incorrect feeding tube positions. The lack of data on feeding tube misposition also prevented the possibility of establishing the specificity of the applied method.

Similar to our results, human studies have reported that evaluating the feeding tube position at the esophageal site allows better detection than that at the gastric site since interference owing to gas interposition in the digestive tract is a great limitation of ultrasound application (22–24). In human medicine, the application of ultrasonography to detect the location of feeding tubes has been evaluated in several studies conducted on critical care or pediatric patients, and it has been proven to have good performance compared with radiographic assessment, which is considered the gold standard method. However, further investigation of the feasibility and applicability of this method is required, and the use of other ancillary techniques is, therefore, recommended (11, 25).

There are numerous advantages of using ultrasound to assess the position of the feeding tube: it is a point-of-care application, allowing the evaluation of patients without excessive movement between medical departments; it avoids radiation exposure of physicians and patients, and it is readily available in the ICU, reducing the delay in starting feeding (13).

This study was conducted in cats and the feeding tubes used had a small size (6 F) and without a stylet. Identification of the tube with ultrasound was relatively easy, using the trachea as a landmark in transverse plane and by moving the probe to the left side to perform the longitudinal plane. The diameter of the tube could also be measured in the longitudinal esophageal scan to compare the measurement with the technical features and to confirm that the structure visible as a double parallel hyperechogenic line was actually the feeding tube.

Another technique used to validate the detection of the tube in the neck and abdominal scans was to inject sterile saline and air, agitated to made microbubbles (bubble contrast), and observe the flow of air bubbles inside the identified structure; however, bubble contrast was not always detected at the esophageal site (detected in 22/25 cats) and it was identified less frequently at the stomach site (detected in 18/25 cats). Statistical analysis of the results derived from the identification of the contrast did not demonstrate the advantage of adding this method to ultrasonographic detection of the feeding tube. If the tube cannot be seen, the use of bubble contrast may help confirm its position in the digestive tract by observing hyperechogenic “fog” produced by the injection of air and liquid in the stomach.

The ultrasound evaluations conducted in this study were performed by veterinarians specialized in ultrasound (RZ, PS, and AD), and the application of this method by emergency veterinarians requires skills to interpret the images and training to perform the evaluation. A limitation of this study was that we did not explore the time necessary for a non-specialist in ultrasound to learn the method and whether the ultrasound operator could influence the diagnostic accuracy of ultrasonography in detecting feeding tube location. Another limitation is the use of two different ultrasound transducers to evaluate the feeding tube position at different sites (the esophagus and stomach). This option allows for better ultrasound performance; however, not all facilities have two different probes available. As previously indicated in the text, no feeding tube was placed in the trachea; therefore, we do not know if ultrasound evaluation could help assess the misplacement of the tube. Veterinarians should be aware that if detection of the feeding tube is not possible through ultrasound, thoracic radiography must be performed to confirm correct placement. When a tube with stylet is applied, the risk of perforation of esophagus could be a complication and a single lateral radiography cannot be enough to differentiate if the tube is in the paraesophageal soft tissue.

## 5 Conclusion

The current study demonstrated that ultrasound is a good method to evaluate nasogastric feeding tube placement at the esophageal site, compared to the radiography, and in addition to the tests already used. Further, identification of the tube at the stomach site is helpful, but may be associate to some false positive. This technique could have several advantages in the daily practice of emergency physicians, however other studies are necessary to assess the time required for an emergency veterinarian, who is not specialized in ultrasound, to acquire the skill and achieve the same accuracy.

## Data availability statement

The original contributions of this study are included in the article. Further inquiries can be directed to the corresponding author.

## Ethics statement

The animal studies were approved by Institutional Ethics and Animal Welfare Committee (protocol no. 1128). The studies were conducted in accordance with the local legislation and institutional requirements. Written informed consent was obtained from the owners for the participation of their animals in this study.

## Author contributions

AG, PS, SR, and RZ: data acquisition. BB and CM: data analysis and interpretation. BB and AB: contributions to conception and design. BB, CM, and SR: writing – original draft preparation. RZ, PS, and AB: review and editing. All authors contributed to the article and approved the submitted version.
